# Age-related alterations in the behavioral response to a novel environment in the African turquoise killifish (*Nothobranchius furzeri*)

**DOI:** 10.3389/fnbeh.2023.1326674

**Published:** 2024-01-08

**Authors:** Valerie Mariën, Ilayda Piskin, Caroline Zandecki, Jolien Van houcke, Lutgarde Arckens

**Affiliations:** ^1^Laboratory of Neuroplasticity and Neuroproteomics, Department of Biology, KU Leuven, Leuven, Belgium; ^2^Laboratory of Developmental Neurobiology, Department of Biology, KU Leuven, Leuven, Belgium; ^3^Leuven Brain Institute, KU Leuven, Leuven, Belgium

**Keywords:** behavior, aging, killifish, open field test, novel tank diving test, locomotion, exploration

## Abstract

The African turquoise killifish (*Nothobranchius furzeri*) has emerged as a popular model organism for neuroscience research in the last decade. One of the reasons for its popularity is its short lifespan for a vertebrate organism. However, little research has been carried out using killifish in behavioral tests, especially looking at changes in their behavior upon aging. Therefore, we used the open field and the novel tank diving test to unravel killifish locomotion, exploration-related behavior, and behavioral changes over their adult lifespan. The characterization of this behavioral baseline is important for future experiments involving pharmacology to improve the aging phenotype. In this study, two cohorts of fish were used, one cohort was tested in the open field test and one cohort was tested in the novel tank diving test. Each cohort was tested from the age of 6 weeks to the age of 24 weeks and measurements were performed every three weeks. In the open field test, we found an increase in the time spent in the center zone from 18 weeks onward, which could indicate altered exploration behavior. However, upon aging, the fish also showed an increased immobility frequency and duration. In addition, after the age of 15 weeks, their locomotion decreased. In the novel tank diving test, we did not observe this aging effect on locomotion or exploration. Killifish spent around 80% of their time in the bottom half of the tank, and we could not observe habituation effects, indicating slow habituation to novel environments. Moreover, we observed that killifish showed homebase behavior in both tests. These homebases are mostly located near the edges of the open field test and at the bottom of the novel tank diving test. Altogether, in the open field test, the largest impact of aging on locomotion and exploration was observed beyond the age of 15 weeks. In the novel tank diving test, no effect of age was found. Therefore, to test the effects of pharmacology on innate behavior, the novel tank diving test is ideally suited because there is no confounding effect of aging.

## 1 Introduction

The African turquoise killifish, *Nothobranchius furzeri*, has an extremely short lifespan for a vertebrate due to its natural habitat. They inhabit temporary ponds in Southeast Africa that fill with water during the rainy season. When the ponds dry out during the dry season, the eggs survive in the soil and only during the next rainy season they will hatch (Terzibasi et al., 2007; Cellerino et al., 2016; Platzer and Englert, 2016). Due to these environmental conditions, killifish have evolved to develop and grow fast, and consequently also age fast. In captivity, killifish start dying of old age between four and six months, and aging characteristics can already be observed by thirtheen weeks of age. Such characteristics include spinal curvature, protrusion of the lip, and loss of coloration in males (Genade et al., 2005; Cellerino et al., 2016; Platzer and Englert, 2016), but also decreased locomotion and cognitive disability (Genade et al., 2005; Valenzano et al., 2006; Terzibasi et al., 2008). Previous research already revealed that killifish experience an age-related loss in the regenerative capacity of the brain, visual system, and fin (Wendler et al., 2015; Van houcke et al., 2021; Vanhunsel et al., 2022). The age-related decline in regeneration potential allows researchers to exploit the killifish to study what hampers regeneration upon aging and to find interventions to boost the regeneration potential (Van houcke et al., 2023). The killifish as a gerontology model can be used in the search for treatments for neurodegenerative diseases, that are characterized by symptoms like impaired movement, memory decline, spatial navigation impairments, anxiety, muscle weakness, etc. To prove the efficacy of treatments for functional recovery from brain damage and age-related neuron loss in the killifish, we need readouts of functional recovery. The open field test is a widely used behavior test to probe for locomotion and anxiety parameters in rodents and has been adapted for use in fish (Hall, 1936; Godwin et al., 2012; Stewart et al., 2014). The reaction of the animal to a novel, open arena is investigated, by analyzing specific exploration and locomotion parameters. Using this test, some similarities between rodent and zebrafish behavior were observed, for example, both display habituation and thigmotaxis behavior (preference for the edges over the center open zone; Stewart et al., 2012). In addition, they also show homebase behavior, where they choose a “safe” space as a return point in the novel arena (Stewart et al., 2010, 2012). The novel tank diving test has been exploited in fish research as an alternative to the open field test (Egan et al., 2009). When zebrafish are placed in the novel tank diving test, they have an initial diving response, where they dive to the bottom of the tank as a sign of anxiety. When treated with nicotine, which has anxiolytic effects, this diving response was not present anymore (Bencan et al., 2009). The novel tank diving test typically studies exploration, diving, and immobility/freezing behavior (Blaser and Rosemberg, 2012; DePasquale et al., 2022). Different teleost species display different behaviors in these types of behavioral tests (Egan et al., 2009; Cachat et al., 2010; Matsunaga and Watanabe, 2010; Godwin et al., 2012; Audira et al., 2021; DePasquale et al., 2022; Lucon-Xiccato et al., 2022; Saiz et al., 2023). In this study, we, therefore, tested the new aging model organism *N. furzeri* in the open field test and the novel tank diving test over its adult lifespan to set a baseline for future behavior experiments.

## 2 Materials and methods

### 2.1 Animals

Fish were housed in 3.5 L aquaria in a ZebTEC multi-linking housing system (Tecniplast). One male was housed with three females until the age of 12 weeks (12w), after which the males were removed. Housing conditions were standardized: a 12/12 h light–dark cycle, water temperature of 28°C, a conductivity of 600 μS, and a pH of 7. Fish were fed twice daily with *Artemia salina* and *Chironomidae* mosquito larvae (Ocean Nutrition). Eggs were collected from breeding pairs as described previously (Van houcke et al., 2021), and golden-eye stage eggs were hatched in a small layer of cold humic acid (1 g/L in system water; Van houcke et al., 2021). Upon hatching, the humic acid was diluted daily for four days with aquarium system water, after which the fish were transferred into the ZebTEC housing system. We tested two groups of 14–16 female African turquoise killifish (*N. furzeri*, inbred GRZ-AD strain) over their adult lifespan in the open field test (*n* = 16) and the novel tank diving test (*n* = 14). Each cohort was tested from the age of 6 weeks to the age of 24 weeks and measurements were performed every three weeks to avoid inter-session habituation. In the open field test, two fish died due to aging between age 12 and 15 weeks. In the novel tank test, Two fish died between ages 18–21 weeks, and two fish died between ages 21–24 weeks. Only female fish were used in this longitudinal experiment to exclude potential variability in the behavior between males and females. All experiments were approved by the KU Leuven ethical committee in accordance with the European Communities Council Directive of 22 September 2010 (2010/63/EU) and the Belgian legislation (KB of 29 May 2013).

### 2.2 Open field test

**Figure 1:**
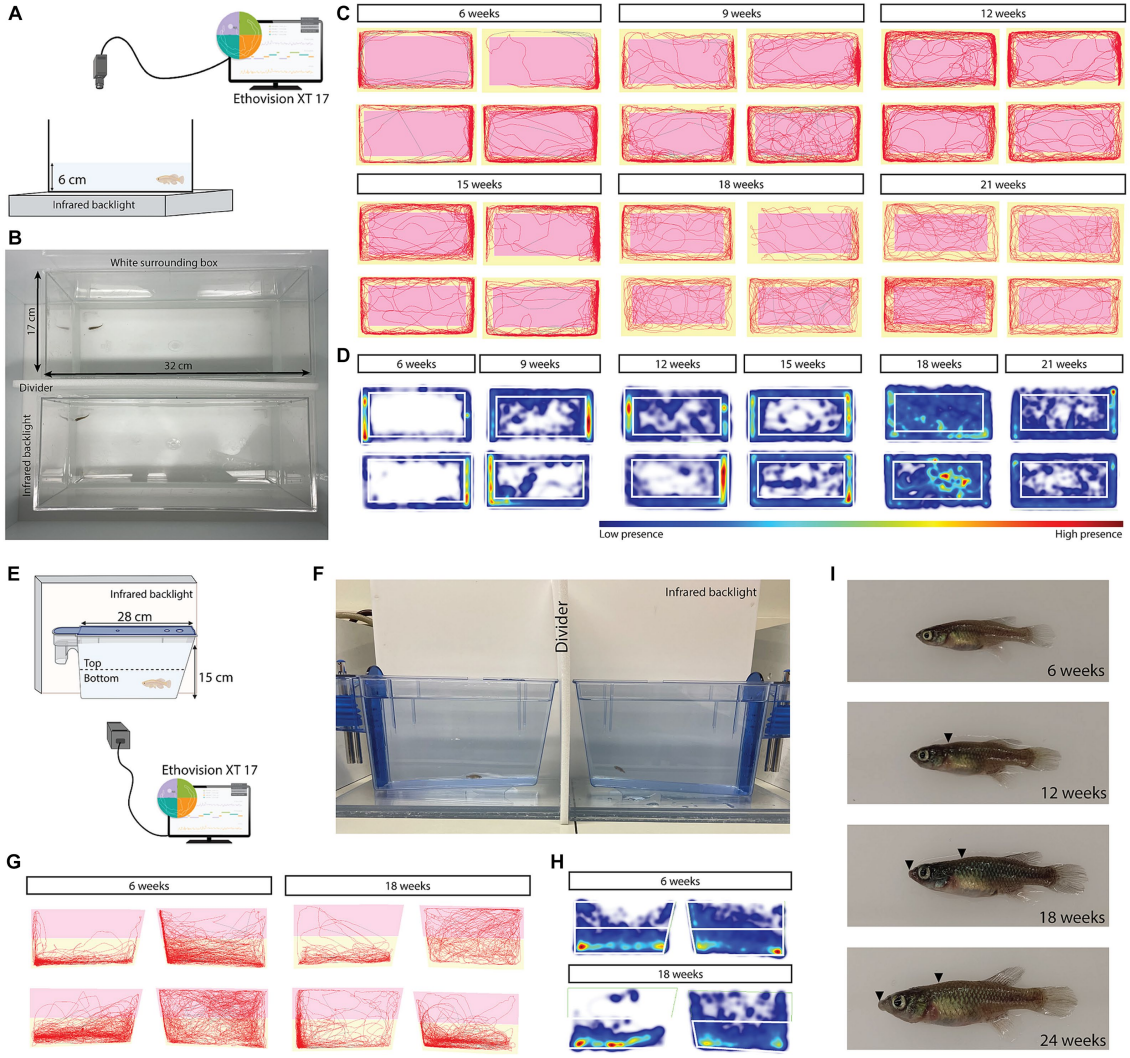
Open field and novel tank diving test setups and representative results of tracks and heatmaps over different ages. Experimental setup for the open field **(A,B)** and novel tank diving test **(E,F)**. Tracks and heatmaps show differences in open field behavior with age, where aged fish spend more time in the center **(C,D)**. The size of the center zone was adjusted to the size (age) of the fish. **(G,H)** Example tracks and heatmaps for the novel tank diving test for 6-week and 18-week-old fish. **(I)** Representative images of 6-, 12-, 18-, and 24-week-old fish. The arrowheads indicate spinal curvature which is visible from 12 weeks and the lip protrusion, which is visible from 18 weeks.

Each fish was tested individually in a transparent tank which was made anti-reflectant with opaque foil on the lowest 6 cm of the tank to prevent the fish from seeing its reflection. The tank (32 cm × 17 cm) was filled with a shallow level (6 cm) of aquarium system water (26°C) to reduce the locomotion in the Z-axis. Animals were transported to the experimental room in their home tanks and netted into the open field test arena. Two fish were recorded at the same time, however, they were not able to see each other because of an opaque partition between the tanks (Figures 1A,B). A white box was placed around the testing arena to make sure there were no influences from the surrounding environment. The arena was virtually divided into a peripheral and a center zone. The size of the center zone was determined for each age, based on the body sizes at different ages (Table 1). The mean body length of the fish age group was subtracted from the size of the peripheral zone to determine the size of the center zone. The tanks were placed on an infrared backlight (λ > 980 nm illumination) and recorded from above with a GigE infrared camera (Basler) at 25 frames per second. In between every recording, the water was changed to exclude compounds of the previous fish and to maintain the same water temperature and oxygenation over all the experiments. The data was recorded, tracked, and analyzed using the Ethovision XT17 software (Noldus Information Technology). All experiments were carried out between 9 a.m. and 4 p.m. The fish were habituated for 5 min, after which 30 min recordings were carried out. To filter tracking noise, a Lowess filter (5 samples), minimal distance moved filter (0.2 cm), and maximum distance moved filter (1 cm) were applied. Different parameters were examined: total distance moved, mean velocity, mean mobility, total time spent in the center zone, total time immobile, and transitions from the periphery to the center (Figures 1C,D). In addition, these variables were analyzed per minute to test for temporal behavior patterns within an age group. Immobility is defined as a velocity lower than 0.1 cm/s for a duration of a minimum of 1 s (Cachat et al., 2010; DePasquale et al., 2022). Mobility means movement of the fish even if the center point remains in the same position. At the age of 9 weeks, we removed two recordings, since the recordings were too short for analysis due to a technical error.

**Table 1:** Mean fish size for each different age group for fish in the open field test.

Fish age	Mean fish size (cm)	SD	SEM	Min (cm)	Max (cm)	Number
6 weeks	1.7	0.23	0.058	1.2	2.0	16
9 weeks	2.1	0.22	0.056	1.8	2.6	16
12 weeks	2.2	0.27	0.068	1.9	2.8	16
15 weeks	2.3	0.27	0.066	1.9	2.8	14
18 weeks	2.5	0.22	0.059	2.1	2.8	14
21 weeks	2.6	0.21	0.056	2.2	3.0	14
24 weeks	2.7	0.20	0.051	2.4	3.1	14

Minimal and maximal length, standard deviation (SD) and standard error of the mean (SEM) of fish per age group and the number of fish measured. The length of the fish per age group was used to determine the size of the center zone.

### 2.3 Novel tank diving test

Animals were tested individually in a 3.5 L ZebTEC aquarium tank (11 cm x 27 cm) filled with aquarium system water to a level of 15 cm. Two tanks were recorded at the same time with an opaque partition between them to prevent the fish from seeing each other (Figures 1E,F). Animals were collected from their home tank and transported in 300 mL glass beakers in an opaque box to the experimental room. Fish were gently poured into the novel tank and recorded for 30 min immediately after being placed in the tank. The arena was virtually divided into a top and bottom zone, each occupying 50% of the water level of the tank. An infrared backlight (λ > 980 nm illumination) was placed behind the tanks and behavior was recorded from the front with a GigE infrared camera (Basler) at 25 frames per second (Figures 1E,F). In between every recording, the water was changed to exclude compounds of the previous fish and to maintain the same water temperature and oxygenation over all the experiments. The data was recorded, tracked, and analyzed using the Ethovision XT17 software (Figures 1G,H) (Noldus Information Technology). All experiments were carried out between 10 a.m. and 4 p.m. To filter out tracking noise, the same filters as in the open field test were applied. Different parameters were analyzed: total distance moved, mean velocity, mean mobility, total time spent in the top/bottom zones, time spent in the lower quadrant, immobility frequency and duration, and latency to the top. In addition, these variables were analyzed per minute to test for temporal behavior patterns within the age group.

### 2.4 Homebase behavior

Homebase behavior was analyzed by virtually dividing the tank into nine zones. The parameters of total distance moved, time spent in each zone (%), and the frequency of visits were calculated using Ethovision XT17. The top three scores for each parameter were determined and indicated on the heatmaps. If a zone scored highest on all three parameters, it was considered a homebase (Stewart et al., 2010).

### 2.5 Statistical analysis

Statistical analysis was performed using GraphPad Prism (v9.3.1). The data was first checked for normal (Gaussian) distribution using the D’Agostino-Pearson omnibus normality test or Shapiro–Wilk test. If conditions were met, a one-way ANOVA was performed followed by Tukey’s multiple comparisons test to compare each age with one another. If the data was not normally distributed, a Kruskal-Wallis test was used followed by Dunn’s multiple comparisons test. 14 to 16 animals were used per age in the open field test and 10 to 14 animals were used per age in the novel tank diving test. All values are presented as the mean ± standard error of the mean (SEM). A cut-off of *p* ≤ 0.05 was used as the threshold for statistical significance. Only statistically significant means are indicated by an asterisk above the bar graphs or next to the line graphs in the habituation figures. To investigate habituation, we examined if the data was normally distributed. If conditions were met, a repeated measures one-way ANOVA was performed per age group. If the data was not normally distributed, a non-parametric Friedman test was used. In the habituation figures, the asterisk is colored in the same color as the age it corresponds with. This indicates that there is a significant effect of testing time for that age group.

## 3 Results

### 3.1 Open field test

#### 3.1.1 Fish size

Because killifish continue to grow during their adult lifespan, we measured their body size at each testing stage to be able to adjust the size of the center zone in the open field test. They grow approximately 1 cm (40%) from the age of 6 weeks to the age of 24 weeks (Table 1). Representative images of a 6, 12, 18, and 24 weeks old fish are visible in Figure 1I.

#### 3.1.2 Locomotion

**Figure 2:**
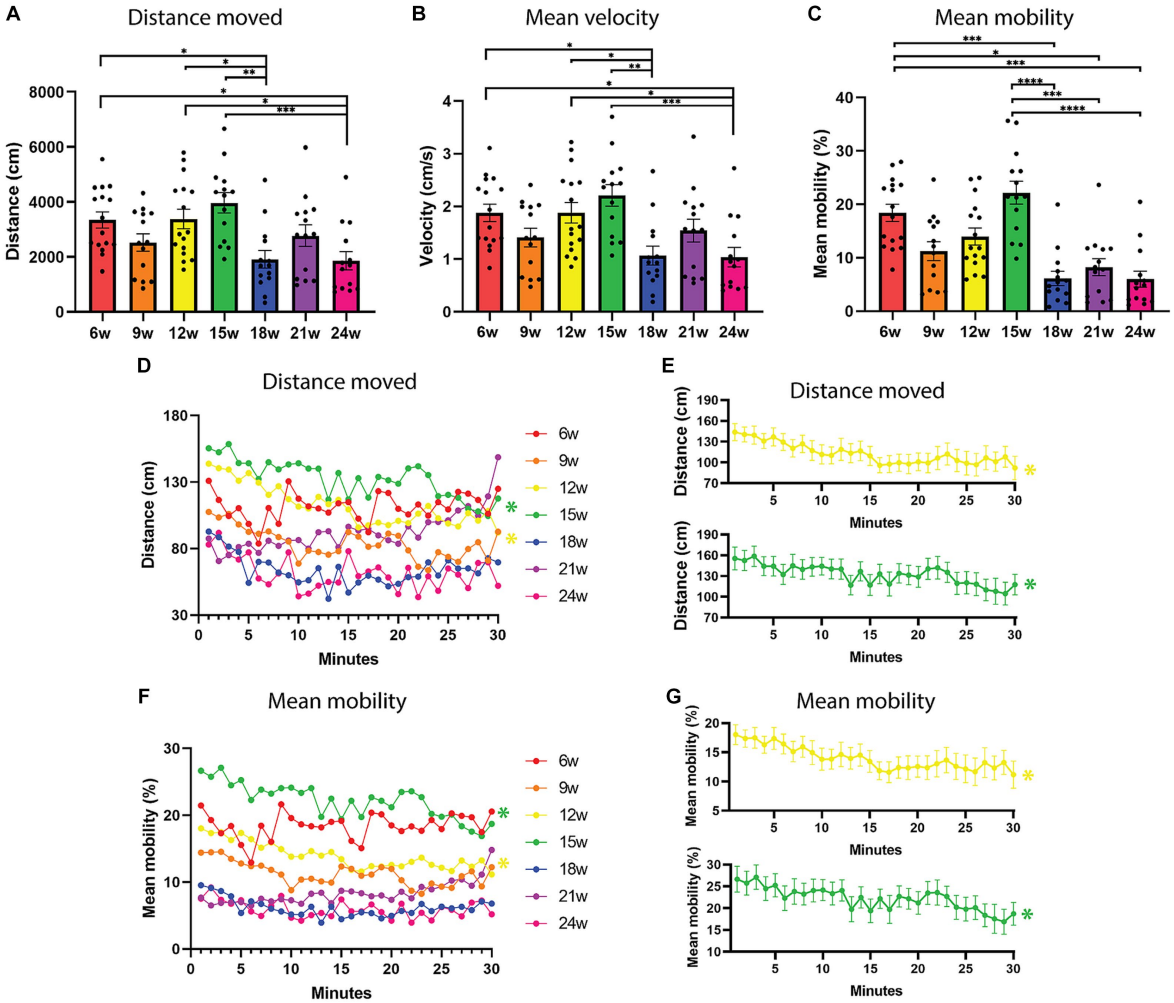
Locomotion parameters in an open field test over the lifespan of the African turquoise killifish. 30-min recordings were carried out and fish were observed in an open field test every three weeks from age 6 weeks until age 24 weeks. **(A)** Total distance moved in a 30-min recording shown for the different ages. **(B)** Mean velocity and **(C)** Mean mobility (movement of the fish even if the center point remains in the same location). **(D,E)** Total distance moved and **(F,G)** mean mobility as a function of fish age and time (1-min time bins) to investigate habituation over time. 12- and 15-week-old fish decreased their distance moved **(E)** and mean mobility **(G)** over the 30-min recording, and hence show habituation (yellow and green lines). Points represent individual fish in panels **(A,B)**. Points represent the means of the different age groups in panels **(C,D)**. *n* = 14–16 per age group. w = weeks. Significant differences are indicated with an asterisk.

Analysis of the total distance moved (averaged over the total group of animals per age; *n* = 14–16) revealed that there was a significant effect of age (*F*_6,95_ = 5.319, *p* < 0.0001, one-way ANOVA) on total distance moved. Using Tukey’s multiple comparisons test, we found significant differences between 6–18 weeks (*p* = 0.0485), 6–24 weeks (*p* = 0.0355), 12–18 weeks (*p* = 0.0404), 12–24 weeks (*p* = 0.0294), 15–18 weeks (*p* = 0.0011), and 15–24 weeks (*p* = 0.0008; Figure 2A). We observed that aged fish swim shorter distances from 18 weeks onward (Figure 2A). Consistent with the total distance moved, the mean velocity showed similar differences (Figure 2B). A one-way ANOVA revealed a significant effect of age on the velocity (*F*_6,95_ = 5.338, *p* < 0.0001). Using Tukey’s multiple comparisons test, we found significant differences between 6–18 weeks (*p* = 0.0425), 6–24 weeks (*p* = 0.0314), 12–18 weeks (*p* = 0.0409), 12–24 weeks (*p* = 0.0301), 15–18 weeks (*p* = 0.0012), and 15–24 weeks (*p* = 0.0008; Figure 2B). Fish were moving with the highest mean velocity at the age of 15 weeks and with the lowest mean velocity at the age of 24 weeks (mean_15w_ = 2.21 cm/s and mean_24w_ 1.04 cm/s, Supplementary Table 1). The mean mobility, which measures when the complete area of the animal is moving even if the center point of the animal is not moving, was the highest at 15 weeks and the lowest at 24 weeks (Figure 2C). A Kruskal-Wallis test was performed and a significant effect of age could be observed for mean mobility (*p* < 0.0001, H = 47.94). Using Dunn’s multiple comparisons test, a significant difference could be observed between 6–18 weeks (*p* = 0.004), 6–21 weeks (*p* = 0.0105), 6–24 weeks (*p* = 0.0002), 15–18 weeks (*p* < 0.0001), 15–21 weeks (*p* = 0.001), and 15–24 weeks (*p* < 0.0001; Figure 2C). In the tracks of the recordings (Figure 1C—all tracks can be found in Supplementary Figure 1), it can be appreciated that the 15-week-old fish moved the most in the open field test. Performing this experiment with an independent cohort of fish at ages 6 and 18 weeks, showed the same pattern of significant differences for distance moved, mean velocity and mean mobility (Supplementary Figures 2A–C).

Taken together, 15-week-old fish are the most mobile, swimming the greatest distance with the highest velocity and 24-week-old fish are the least mobile, swimming the least and with the lowest mean velocity (all tracks of all fish can be found in Supplementary Figure 1).

#### 3.1.3 Habituation of locomotion

We also investigated the temporal trend of these parameters per age group, to look for habituation to the open field test. To this end, the data were represented as 1 min time bins over the 30 min recordings (Figures 2D–G). Using repeated measures one-way ANOVA, we found a significant effect of time on distance moved for 12 weeks (*p* = 0.0030) and 15 weeks (*p* = 0.0343), where the distance moved decreased over time (Figures 2D,E, yellow and green lines). The same temporal changes were observed for the mean velocity, a significant effect of time was found for 12 weeks (*p* = 0.0028) and 15 weeks (*p* = 0.0355), where the velocity decreased over time (repeated measured one-way ANOVA, data not shown since correlated to distance moved). Lastly, this temporal trend was again observed for the ages 12 weeks (*p* = 0.0161) and 15 weeks (*p* = 0.0333) for mean mobility, where the mobility decreased over the 30 min recording (repeated measures one-way ANOVA, Figures 2F,G).

#### 3.1.4 Exploration-related behavior

**Figure 3:**
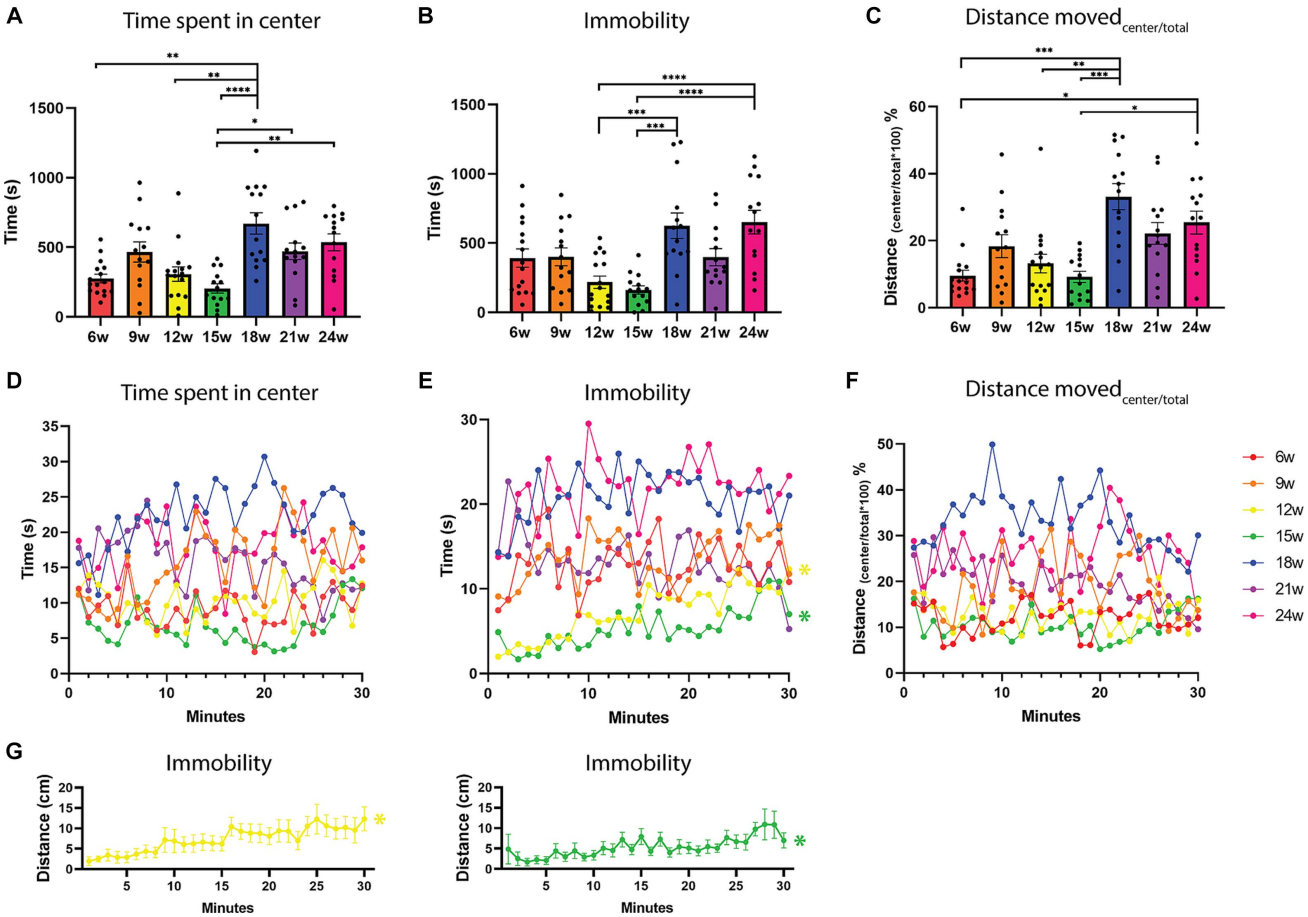
Exploration-related parameters in an open field test over the lifespan of the African turquoise killifish. 30-min recordings were carried out and fish were observed in an open field test every three weeks from the age of 6 weeks until the age of 24 weeks. **(A)** The total time spent in the center zone during a 30-min recording for the different ages shows that upon aging, fish spent more time in the center. **(B)** The total duration of immobility, and **(C)** the distance moved in the center divided by the total distance moved as a function of age. With age, fish moved a greater distance in the center zone. Time spent in the center **(D)**, immobility **(E)**, and the percentage distance moved in the center **(F)** as a function of fish age and time (1-min time bins). **(E,G)** Habituation was only observed for the 12- and 15-week-old fish, where immobility increased over time (yellow and green lines). Immobility is defined as a velocity lower than 0.1 cm/s for a duration of at least 1 s. **(F)** The distance moved in the center divided by the total distance moved, shows the percentage of distance moved in the center. Points represent individual fish in panels **(A–C)**. Points indicate means of the different fish per age group in panels **(D–G)**. *n* = 14–16 per age group. w = weeks. Significant differences are indicated with an asterisk. ^*^p < 0.05, ^**^p < 0.01, ^***^p < 0.001, ^****^p < 0.0001.

Time spent in the center zone is typically used to describe the anxiety and exploration levels of rodents and zebrafish (Godwin et al., 2012). More time spent in the center zone is interpreted as being less anxious and more exploratory. Using a Kruskal-Wallis test, a significant effect of age on the time spent in the center zone was found (H = 35.82, *p* < 0.0001). We observed a clear increase in time spent in the center zone from 18 weeks onward (Figures 1D, 3A; Supplementary Figure 3).

In addition, we observed a significant effect of age on the duration that fish were immobile, which is defined as moving less than 0.1 cm/s for at least 1 s [one-way ANOVA, *F*(6, 95) =7.729, *p* < 0.0001]. From 18 weeks onward, fish spent significantly more time immobile compared to ages 12 and 15 weeks. Tukey’s multiple comparisons test showed a significant difference for 12–18 weeks (*p* = 0.0005), 12–24 weeks (*p* = 0.0002), 15–18 weeks (*p* < 0.0001), and 15–24 weeks (*p* < 0.0001; Figure 3B), in which the older ages showed more immobility. A trend between 6 and 24 weeks in increased immobility was also observed, where the older fish are less mobile (*p* = 0.0801). In addition, we found that aged fish had a higher frequency of being immobile [6–24 weeks (*p* = 0.0376), 9–24 weeks (*p* = 0.0452), 12–18 weeks (*p* = 0.0003), 12–24 weeks (*p* < 0.0001), 15–18 weeks (*p* < 0.0001), and 15–24 weeks (*p* < 0.0001, Supplementary Figure 4A)]. For zone transitions from the peripheral to the center zone, no differences were observed with aging (Supplementary Figure 4B).

From 18 weeks onward, the fish swim shorter distances (Figure 2A), and they spend more time in the center (Figure 3A). In addition, we observed a longer duration that aged fish were immobile (Figure 3B). We next wondered if the fish spent more time in the center because they were more immobile in the center. Therefore, we calculated the distance moved in the center divided by the total distance moved and here we noticed that the percentage of distance moved in the center was also higher for aged fish. More specifically, we observed the biggest difference between 6–18 weeks (*p* = 0.003), 6–24 weeks (*p* = 0.0215), 12–18 weeks (*p* = 0.007), 15–18 weeks (*p* = 0.002), and 15–24 weeks (*p* = 0.0162; One-way ANOVA, Kruskal-Wallis test, Figure 3C). So, aged fish do not freeze more in the center, on the contrary, percent-wise they move more in the center zone compared to younger fish (Figure 3C; Supplementary Figure 4C). Performing this experiment with an independent cohort of fish at ages 6 and 18 weeks, showed the same pattern of significant differences for time spent in the center, immobility and distance moved in the center compared to the total distance moved (Supplementary Figures 2D–F).

#### 3.1.5 Habituation of exploration

No habituation effect over time was observed in the time spent in the center and the percentage distance moved in the center (Figures 3D,F, one-way repeated measures ANOVA). It seems that the fish switch between periods of being in the center alternating with periods in the periphery (Figure 3D). A habituation effect over the 30-min recording was observed for immobility for the ages 12 and 15 weeks, in which the time immobile increased over the 30 min recording within the age group (*p* < 0.0001 for both, non-parametric one-way repeated measures ANOVA; Friedman test; Figures 3E,G, yellow and green lines). However, overall the 12- and 15-week-old fish spend the least of their time immobile, are the most active, and spend the least time in the center zone (Figures 2, 3A,B).

### 3.2 Novel tank diving test

#### 3.2.1 Locomotion

**Figure 4:**
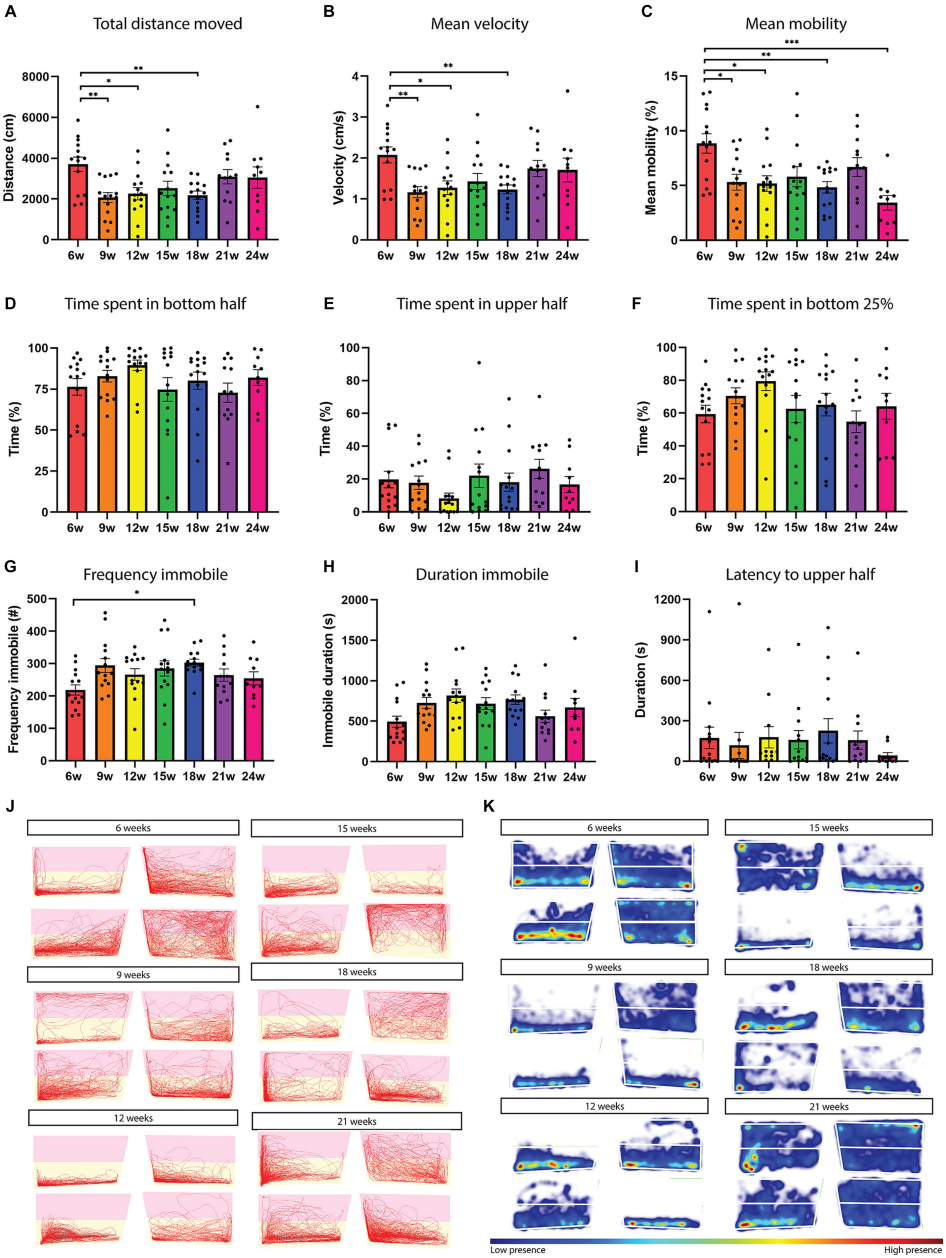
Locomotion and exploration-related parameters in the novel tank diving test do not reveal any clear aging effect. Total distance moved **(A)**, mean velocity **(B)**, and mean mobility **(C)** as a function of age. **(D)** Time spent in the bottom half **(E)** upper half, and **(F)** bottom 25% in function of the different fish ages. **(G)** Immobility frequency and duration **(H)** as a function of fish age. **(I)** Latency to reach the upper half as a function of fish age. Immobility is defined as a velocity lower than 0.1 cm/s for a duration of at least 1 s. **(J,K)** Representative traces and heatmaps of different fish ages over the 30 min recording. Pink indicates the upper half and yellow the bottom half. High presence is indicated in red in the heatmap. *n* = 10–14 per age group. w = weeks. Significant differences are indicated with an asterisk. ^*^p < 0.05, ^**^p < 0.01, ^***^p < 0.001.

A difference in total distance moved and mean velocity (Figures 4A,B) was observed between 6–9 weeks (*p* = 0.0084, *p* = 0.0080), 6–12 weeks (*p* = 0.0294, *p* = 0.0295), and 6–18 weeks (*p* = 0.0184, *p* = 0.0182). Six-week-old fish are the most mobile [6–9 weeks (*p* = 0.212), 6–12 weeks (*p* = 0.0148), 6–18 weeks (*p* = 0.0054), and 6–24 weeks (*p* = 0.0002); Figures 1G,H, 4C], they swim with the highest mean velocity and move the biggest distance (Figures 4A,B,J,K). The 24-week-old fish are the least mobile (Supplementary Table 2). No clear aging effect was observed in the locomotion parameters of the novel tank diving test. When analyzing the habituation behavior over the 30 min recording, we found that only 21-week-old fish increased the distance moved over the recording time (Mixed-effects ANOVA, *p* = 0.0254, Supplementary Figures 5A,G, indigo line). All tracks of the recordings can be found in Supplementary Figure 6.

#### 3.2.2 Exploration-related behavior

Killifish showed a clear preference for the bottom half of the tank at all ages of their lifespan. Approximately 80% (Supplementary Table 2) of their time was spent in the bottom half of the tank (Figures 4D,J,K). No differences were observed between the ages in the time spent at the top/bottom of the tank (Figures 4D,E). When examining the time spent in the bottom 25% of the tank, no age-related differences were observed (Figure 4F). Immobility frequency and duration also did not alter significantly upon aging. Only a difference was detected between 6- and 18-week-old fish in the frequency of immobility, where the aged fish were more frequently immobile (*p* = 0.0315, one-way ANOVA; Figures 4G,H). Latency to the upper half and frequency of transitions to the upper half also did not differ among the age groups (Figure 4I and not shown). All heatmaps of the recordings can be found in Supplementary Figure 7. We next investigated the temporal pattern of behavior by representing the data in 1 min time bins (Supplementary Figure 5). Here we observed an effect of testing time for the time spent in the top for ages 6, 15, and 21 weeks, where fish increased their time spent in the upper half of the tank after 5–10 min (non-parametric repeated-measures one-way ANOVA, Friedman test, *p* = 0.0361, *p* = 0.0359, and *p* < 0.0001 respectively; Supplementary Figures 5E,H, red, green, and indigo colored lines).

### 3.3 Homebase behavior

**Figure 5:**
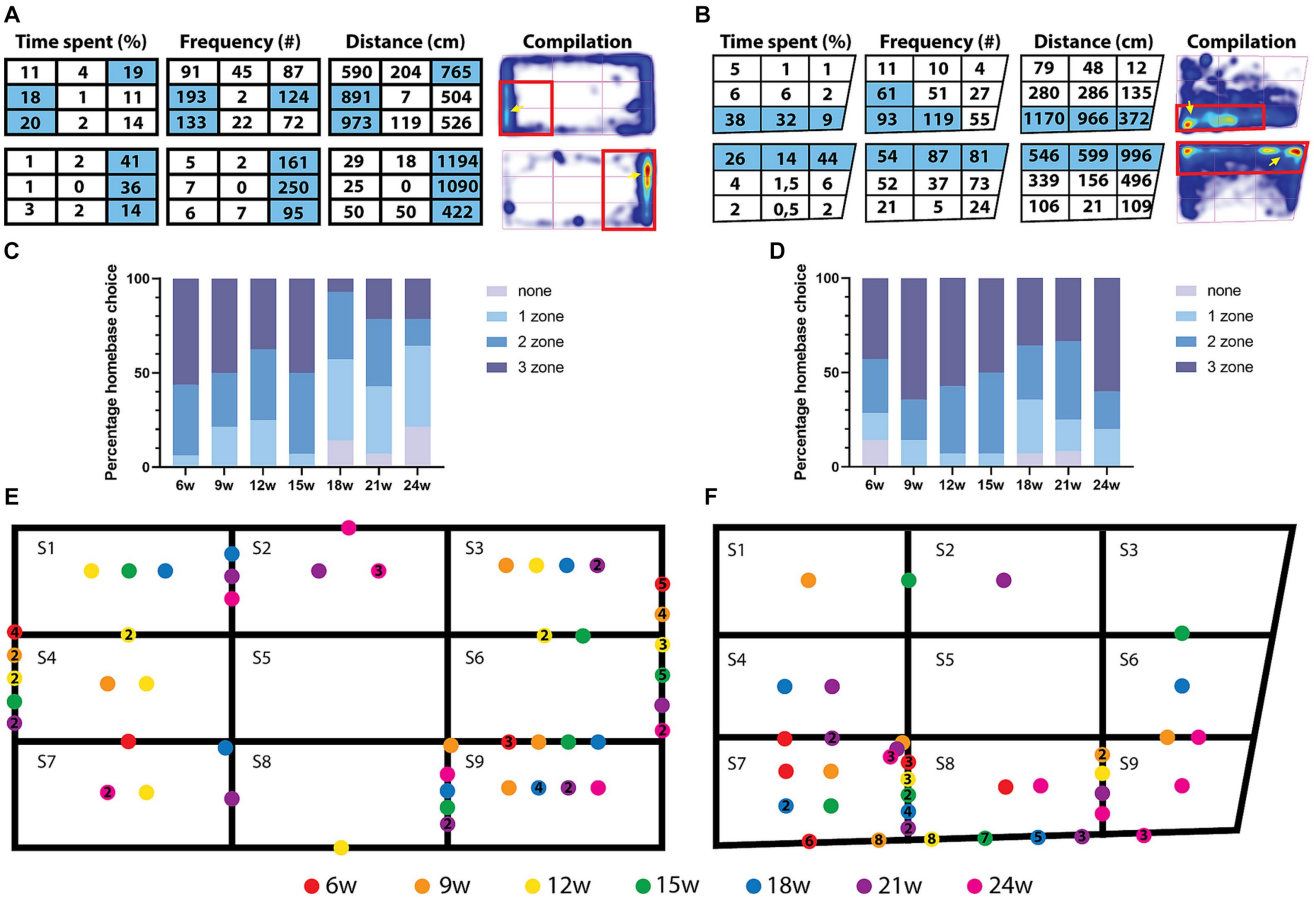
Homebase choice in the open field and novel tank diving test. **(A,B)** Parameters used to determine homebase choice are time spent in a zone, frequency of visits to a zone and distance moved in a zone. **(A)** Example of two fish in the open field test and **(B)** novel tank diving test. **(C,D)** Percentages of homebase choice. **(E,F)** Summary scheme of homebase behavior in the open field **(E)** and novel tank diving test **(F)**. **(A,B)** Homebase behavior was determined on the top three scores for three different parameters: Time spent (%) in a zone, frequency of visits to that zone and distance moved in that zone. If a zone has the highest score for all three parameters, it is considered a homebase. The heatmaps show the time spent in a certain zone, and the homebase is indicated with a red rectangle. Yellow arrows indicate the hotspots in the heatmaps, which are located in the homebase. **(C)** Percentage homebase choice in the open field test, note that upon aging, the 3-zone homebase is much less frequent, while the 1-zone homebase is much more frequent. **(D)** Percentage homebase choice in the novel tank diving test. **(E,F)** Each homebase is indicated as a dot where the color corresponds to the fish’s age. Often, a 3-zone homebase was established (indicated as dots on the outer border, meaning that they use the three zones on that side of the tank as a homebase). Both tanks were divided into nine zones (S1–S9). The figures on the colored dots indicate the number of animals of that age that have their homebase on that location. When a dot is placed on a line between two zones, it means that the fish has a 2-zone homebase. A dot in the corner of the 3 zones indicates an L-shaped homebase.

A homebase is defined as a preferred area where the animals frequently return to and spend the most time. This has been described previously in rodents, where rats show the highest grooming and rearing activities in their homebase (Eilam and Golani, 1989). Homebase behavior was investigated in zebrafish by measuring the time spent (%), distance moved (cm), and frequency of visits after dividing the arena into nine equal-sized zones (Stewart et al., 2010; Figure 5). If a zone scores the highest on all three of these parameters, it is considered a homebase (Figures 5A,B). We applied this same strategy and found that most fish had a homebase except for six fish in the open field test (18 weeks: *n* = 2; 21 weeks: *n* = 1; 2 4 weeks: *n* = 3) and four fish in the novel tank diving test (6 weeks: *n* = 2; 18 weeks: *n* = 1; 21 weeks: *n* = 1; Figures 5C,D). Some fish also had a non-adjacent homebase, meaning that they chose two zones further away from each other as preferred zones, e.g., S4 and S6 (Figure 5E; Open field test—6 weeks: *n* = 2; 9 weeks: *n* = 3; 12 weeks: *n* = 2; 15 weeks: *n* = 4; 18 weeks: *n* = 2 vs. Novel tank diving test—12 weeks: *n* = 2; 15 weeks: *n* = 2). These are not indicated in the summary scheme (Figures 5E,F). The homebase in the open field test was always located near the walls, and never in the center of the tank (note the absence of a dot in S5 in the scheme; Figure 5E). In the open field test, combining all ages, 26% of the fish had a 1-zone homebase, 33% had a 2-zone homebase, and 35% had a 3-zone homebase, 6% of the fish had no homebase. When plotting the percentages of homebase-size per age, we observe that in the open field test, upon aging, the homebase becomes smaller (Figure 5C). In 6-week-old fish, 56% of fish had a 3-zone homebase and 6% a 1-zone homebase. In 24-week-old fish, 21% had a 3-zone homebase and 43% had a 1-zone homebase. In the novel tank diving test, when combining all ages, we observed that most fish had their homebase in the bottom 3 zones (92%, Figure 5F). Only 4 fish established a homebase in one of the upper three zones, and 14 fish established a homebase in the middle three zones of the tank. Combining all ages, 15% of the fish had a 1-zone homebase, 32% a 2-zone homebase, and 49% a 3-zone homebase, 4% had no homebase. When plotting the percentages of homebase-size per age, no effect of age on the size of the homebase was observed in the novel tank diving test.

## 4 Discussion

In this study, we investigated killifish behavior over their adult lifespan in an open field and novel tank diving test to set a baseline for future behavioral experiments. The open field test and the novel tank diving test have been widely used by zebrafish and medaka researchers in the last decade (Egan et al., 2009; Cachat et al., 2010; Matsunaga and Watanabe, 2010; Rosemberg et al., 2011; Godwin et al., 2012; Stewart et al., 2012, 2014; Liu et al., 2018; Lucon-Xiccato et al., 2020, 2022; Audira et al., 2021). Since killifish are fast-aging, it is important to investigate behavioral changes over their adult lifespan, as this might influence experiments where the effect of a certain drug on behavior over different ages is studied (Thoré et al., 2023). Previous experiments in killifish were carried out on different strains or at different life stages with different recording times (Genade et al., 2005; Valenzano et al., 2006; Thoré et al., 2018, 2019, 2020, 2023; Evsiukova et al., 2021). In the study by Evsiukova and colleagues, the ZMZ1001 strain was used in the novel tank diving test with recordings of 5 min (Evsiukova et al., 2021). Thoré and colleagues used the novel tank diving test in the MZCS-222 strain, with 10 min recordings and a frequency of 5 recordings per week (Thoré et al., 2023). The study by Genade and colleagues used the same strain as in our experiments (GRZ) for conducting open field tests, and measured the fish weekly from 5 to 9 weeks of age (Genade et al., 2005). Similarly, Valenzano and colleagues used the GRZ strain in the open field test and tested the fish from 5 to 9 weeks of age (Valenzano et al., 2006). However, in these open field studies, the fish were recorded for 5 min (Genade et al., 2005), which is clearly different from our study in which we recorded for 30 min. In addition, in our study, for the first time, longitudinal measurements are performed over the entire adult killifish lifespan in both the open field test and the novel tank diving test.

### 4.1 Locomotion

In the open field test, we observed that the total distance moved and mean velocity were highest at 15 weeks of age and decreased at the older life stages (18 and 24 weeks). This indicates that killifish are the fittest and most active at 15 weeks. Indeed, mean mobility was also the highest at this time point, although 6-week-old fish had a similar mean mobility and moved similar distances. Mobility is defined as movement even if the center point of the animal is not moving, i.e., rotation of the fish or small fin movements. These observations are not in line with what was previously found in killifish, where a decline in velocity and time moving was observed from 6 to 9 weeks of age (Valenzano et al., 2006). In that study, five males and five females were used, and males and females are known to have different behaviors (Philpott et al., 2012; Evsiukova et al., 2021). In our study, we used 14 female fish, and each fish was housed with two other females and one male. In the study by Valenzano, killifish were housed together in groups of 20 fish, which might also explain the difference with our results (Parker et al., 2012). Genade and colleagues also observed a decrease in the time spent moving from 5 to 9 weeks of age (Genade et al., 2005). These observations could also be explained by the fact that the fish are recorded every week in both the study by Valenzano and colleagues and Genade and colleagues. This might induce inter-session habituation. To avoid inter-session habituation effects, we only performed our measurements every three weeks. In rodent open-field tests, there is an inter-session habituation, where mice decrease their locomotion upon repeated testing (daily for four consecutive days, every four days, eight-day intervals; Bothe et al., 2004; Kasahara et al., 2007; Chen et al., 2023). Also in a rodent forced-swim test, it has been described that upon testing repeatedly, the immobility time increases. Here, they recommend an inter-test interval of more than one week (Cnops et al., 2022). This inter-session habituation has previously been described in medaka as well (Matsunaga and Watanabe, 2010).

A decline in locomotion is only observed in the open field test from the age of 18 weeks onward, where a clear decrease in mean mobility, total distance moved, and mean velocity are observed compared to the younger ages in the open field test. This decline in locomotion due to aging has previously been described in many different species like zebrafish, rats, mice, and humans (Gilbert et al., 2014). In the novel tank diving test, we did not observe this aging effect on locomotion. Only the 6-week-old fish were significantly different from older ages [9, 12, 18 (24 weeks only mean mobility)]. The differences in distance moved in the open field vs. the novel tank test are striking and might be explained by different factors. It might be due to the 3D movements in the novel tank diving test, which are only measured in 2D. In the open-field test, the fish are limited in the Z-axis since they only have a shallow water level (6 cm), so the open-field test might be more accurate for quantifying locomotion parameters. In the future, recordings in 3D will be helpful in more accurately measuring the locomotion in the novel tank diving test (Cachat et al., 2011). Another reason could be that the fish in the open field test had an acclimation period of 5 min before the recording started and the fish in the novel tank test did not. Overall, less distance was moved in the novel tank test compared to the open field test for ages 9, 12, 15 weeks, and more for ages 6, 18, 21, and 24 weeks. This might indicate that the 6-, 18-, 21-, and 24-week-old fish move more in the Z-axis and less in the XY-axis. Additionally, the fish in the open field test were transported in their home tank to the behavior room and netted into the open field test. In the novel tank diving test, the fish were transported in glass beakers and poured into the novel tank. These different ways of transportation might also influence their behavior and internal state. Future experiments will have to repeat this experiment and compare the locomotion in the open field an novel tank diving test to confirm the actual reason for these differences.

In addition, we only used one cohort of fish per test. Ideally, this experiment should be repeated with multiple cohorts to confirm these results. We therefore performed an independent experiment in the open field test with only the ages 6 and 18 weeks (Supplementary Figure 2). Here, we already observed the same statistical effects (unpaired *t*-test), which already partially confirms our results.

### 4.2 Exploration

In the open field test, we observed the same thigmotaxis behavior as found in other fish species (Stewart et al., 2012; Ahmad and Richardson, 2013; Lucon-Xiccato et al., 2022; Saiz et al., 2023). This means that killifish avoid the center zone and prefer swimming close to the edges. In rodents, goldfish and zebrafish, spending more time in the peripheral zone is associated with increased anxiety. Using pharmacology, this anxiety decreases and the animals will spend more time in the center (Prut and Belzung, 2003; Schnörr et al., 2012; Johnson and Hamilton, 2017; Chen et al., 2023). The center zone is more open and exposed and therefore associated with more risk for predators. This center avoidance was constant over testing time, as observed in zebrafish (Baiamonte et al., 2016; Lucon-Xiccato et al., 2022). Remarkably, the thigmotaxis behavior decreased in older killifish. From 18 weeks onward, the fish spent more time in the center and moved a greater distance in the center. This might indicate an increase in exploration in older fish. However, we also found an increase in immobility/freezing duration (when fish move less than 0.1 cm/s for at least 1 s) in the older ages (21 and 24 weeks compared to 12 and 15 weeks), which would indicate decreased exploration. Freezing has been described previously in other fish species as a result of an electric shock or placement in the novel tank diving test (Cachat et al., 2010; Duboué et al., 2017). When analyzing the frequency of freezing/immobility, there was no effect of age, except for the 21- vs. 24-week-old fish, where the 21-week-old fish froze more frequently, but for shorter times, since their total immobility time was lower. Interestingly, similar to what Thoré and colleagues described, we also found that the most active killifish (greatest distance moved—15-week-old fish), spent more time in the periphery and so are more risk-averse (Thoré et al., 2018). To know if this increased/decreased exploration is related to anxiety, we would have to use behavioral tests with anxiolytic compounds to ascertain the anxiety-like nature of the thigmotaxis behavior in killifish.

Other parameters used to potentially describe anxiety are the distance moved and the time spent moving (Lucon-Xiccato et al., 2022). Animals with higher anxiety show lower activity and slow habituation over time (Godwin et al., 2012; Kotrschal et al., 2014). We did not detect a lot of habituation over time for the killifish, in the older ages, i.e., 18, 21, and 24 weeks, no habituation was observed in the open field test. This strengthens the assumption that killifish do become less explorative upon aging and that the increase in time spent in the center zone does not reflect an increased exploration/decreased anxiety. In addition, aged fish also did not establish a homebase in the center of the open field test. There might be other factors at play from 15 weeks onward that could explain why killifish are less averse to the center zone. These could be, vision loss, but we would then expect a more gradual pattern since a decrease in visual acuity happens gradually from 6 weeks onward (Vanhunsel et al., 2021). Another reason could be that they lose their lateral line function, and therefore have problems orienting near the edges of the tank. Hair cell aging in the lateral line has been shown already, as well as the fact that younger hair cells have longer survival (Lukasz et al., 2022). It has already been shown that zebrafish can regenerate their lateral line hair cells, even in older fish (Pinto-Teixeira et al., 2015). Yet, killifish lose their regeneration capacities upon aging in their brain, visual system, and fin (Wendler et al., 2015; Van houcke et al., 2021; Vanhunsel et al., 2022; Örling et al., 2023). Therefore, it might be that killifish have lateral line dysfunctions upon aging as well. In addition, moving and freezing bouts have a fluctuating pattern in our results, as was also observed in zebrafish. This suggests that killifish, like zebrafish, alternate between phases of lower and higher exploration in a novel environment (Stewart et al., 2012). A decrease in anxiety has already been observed upon aging in rats (Torras-Garcia et al., 2005). Contradictorily, an increase in anxiety and decreased exploration with aging has also been observed in rats (1 year) and zebrafish. Aged zebrafish (>18 months) spend more time on the bottom of the novel tank diving test (Furchtgott et al., 1961; Kacprzak et al., 2017; Lomidze et al., 2020).

In the novel tank diving test, we could not detect any clear exploration changes upon aging. All the fish at different ages spent around 80% of their time in the bottom half of the tank. Moreover, when investigating the temporal pattern of behavior, we observed an increase in the time spent in the top zone for some of the ages (6, 15, and 21 weeks) indicating habituation to the novel tank and increasing exploration. However, this was not observed for all the different ages. Another study on killifish (ZMZ1001 strain) used the novel tank diving test (5 min) and showed that aged male killifish (6 months old) stayed closer to the bottom than younger males. For the females, no difference was observed between the different ages in the distance moved from the bottom or the time spent in the lower third of the tank (Evsiukova et al., 2021). To test if killifish stay close to the bottom because they are anxious, anxiolytic drugs could be added to confirm this. If they would then spend more time in the top, the novel tank diving test can be useful for investigating the effect of drugs on anxiety over different ages of the killifish lifespan (Stewart et al., 2015). This way, we can ensure that the observed effect is due to the drugs and not due to changes in baseline behavior upon aging.

In addition, we found that killifish show homebase behavior. Homebase behavior is described as a place in the test arena that is preferred by the animal, it is a place where the animals frequently return to and spend a longer time. This has already been described in zebrafish and rodents (Eilam and Golani, 1989; Stewart et al., 2010). In the open field test, killifish preferred the shorter sides of the tank (30% of the fish) as a homebase. The homebase was always found close to the walls in the open field test and the majority of the time, close to the bottom in the novel tank diving test. This indicates that fish chose a place where they feel safe as a homebase. In the open field test, it seems that with aging, the homebase becomes smaller (1-zone) or is lost, 73% of the 1-zone homebases in the open field test belong to the ages 18, 21, and 24 weeks. All the fish (*n* = 6) without a homebase are from these ages as well. Only 20% of the 3-zone homebases belong to the ages 18, 21, and 24 weeks. In the novel tank diving test, most fish have a 3-zone homebase at the bottom of the tank.

### 4.3 Comparison of killifish behavior with zebrafish and medaka

**Figure 6:**
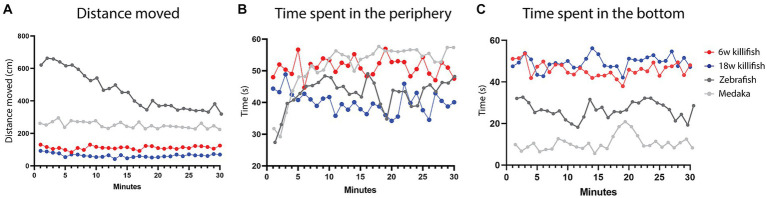
Comparison in total distance moved in the open field test **(A)**, time spent in the periphery in the open field test **(B)**, and time spent in the bottom half of the novel tank diving test between young (6 weeks, red) and old (18 weeks, blue) killifish and zebrafish (dark gray) and medaka (light gray). Killifish move a much smaller distance than zebrafish and medaka **(A)** and initially spend more time in the periphery than zebrafish and medaka **(B)**. **(C)** Comparison of time spent in the bottom half of the novel tank diving test with zebrafish and medaka, shows that killifish spent most of their time in the bottom half of the tank, which is much more than medaka and zebrafish [zebrafish and medaka data reused from Lucon-Xiccato et al. (2022)—Creative Commons—http://creativecommons.org/licenses/by/4.0/].

When we compare our data of the open field test with zebrafish and medaka, we observe that killifish move a much smaller distance in a 30-min recording [Figure 6A—Data zebrafish and medaka reused from Lucon-Xiccato et al., 2022]. They move approximately 25% of the distance of zebrafish and 50% of the distance of medaka. For zebrafish, a habituation effect is observed over time, where they decrease locomotion and exploration within a 30-min recording. In contrast, this effect is not observed for medaka. For killifish, we only observed a habituation effect for ages 12 and 15 weeks, where a decrease in distance moved was observed over the 30-min recording. For the other ages, no habituation was observed in the distance moved similar to medaka. Apart from the initial habituation in medaka (first 5 min), the time that young killifish spent in the periphery is similar to medaka. Old killifish showed a similar pattern to zebrafish after the initial 5 min [Figure 6B—Data zebrafish and medaka reused from Lucon-Xiccato et al., 2022]. While zebrafish and medaka initially (first 10 min) spend more time exploring the center zone and afterward stay close to the edges of the open field arena [Figure 6B—Data zebrafish and medaka reproduced from Lucon-Xiccato et al., 2022], killifish preferred to stay at the edges in the beginning and only later on started exploring the center. This is mostly the case in older killifish (18 weeks and older). Young killifish tended to stay close to the edges over the total recording. In the novel tank diving test, we observed that killifish preferred spending most of their time (approximately 80%) in the bottom half of the tank. This makes killifish very different from both zebrafish and medaka. Medaka spend most of their time in the top half of the tank and only spend around 20% of their time in the bottom half of the tank. Zebrafish spend around 50% of their time in the bottom of the tank (Figure 6C). In future experiments, longer recordings of up to 4 h could be performed to detect if killifish show slow habituation, as was observed for some medaka studies (Lucon-Xiccato et al., 2022).

Comparing data between teleost species needs to be done carefully, because there are small differences in the setups. Stewart and colleagues showed that the size of the open field test arena influences the scale of locomotion behavior but not the temporal behavior pattern in zebrafish (Stewart et al., 2012). Since the size of our open field arena (32 × 17cm) is smaller than the one of Luccon-Xiccato and colleagues (40 × 40 cm), this might be an explanation for why killifish swim much less. However, after analysis of *in-house* data in bigger tanks (49 × 19cm), we did not find a correlation between bigger tanks and higher locomotion for the killifish (data not shown). This is important for other behavioral experiments such as learning and memory tests where fish have to reach a certain area to associate a certain cue/color/stimulus with a reward/aversive stimulus. If we use the same tank size as in zebrafish research, it might take killifish too long to reach a certain area and therefore to learn to associate a certain area/cue/color/stimulus with a positive reward or negative stimulus. Therefore, it might be better to use smaller mazes for these kinds of experiments using killifish in the future.

In the novel tank diving test, we expected that killifish also spend most of their time in the top of the tank, since they live in shallow, temporary ponds like medaka. Therefore, the fact that they spent most of their time in the bottom half of the tank might indicate anxiety, and it might be that killifish have slow habituation, therefore not increasing the time in the top zone over the 30-min recording. It has been shown in several aquatic species that they dive to the bottom to avoid risk (Doran et al., 2022). Evsiukova and colleagues also observed that killifish spent around 80% of their time in the bottom third of the tank (Evsiukova et al., 2021). Thoré and colleagues observed that adult killifish (MZCS-222 strain) spent more time in the lower 25% of the novel tank test than juvenile killifish. However, they observed that the adult killifish spent approximately 33% of their time in the bottom 25% of the tank (Thoré et al., 2023). This could be an effect of the testing time (10 vs. 30 min) or the different strain (MZCS-222 vs. GRZ-AD). Another reason for a decrease in the time spent in the bottom could be the habituation of the fish in the study of Thoré and colleagues since the fish were tested five times a week for ten weeks (Thoré et al., 2023).

Overall, killifish showed clear thigmotaxis behavior in the open field test from the age of 6 weeks to the age of 15 weeks. Aged fish (18 weeks and older) still spent more than 50% of the time near the edges of the tank. Future experiments using anxiolytic drugs should be performed to confirm that spending time in the center really indicates a decrease in anxiety. Unexpectedly, in the novel tank test, there was not much exploration of the upper part of the tank. It also seems that killifish show no or slow habituation in these kinds of tests. Therefore, we should examine longer testing times to uncover if there would be habituation. In the novel tank diving test, anxiolytic drugs should be applied to track if killifish explore the top half of the tank due to decreased anxiety. In addition, since killifish move much smaller distances than zebrafish an medaka, smaller mazes should be used in killifish behavioral tests. To further characterize killifish behavior and anxiety, more behavioral tests should be explored over the adult lifespan, like for example the light/dark test and the active avoidance test. Future studies should also make use of 3D measurements of killifish behavior to fully understand their spatial behavior.

## Data availability statement

The original contributions presented in the study are included in the article/Supplementary material, further inquiries can be directed to the corresponding author.

## Ethics statement

All experiments were approved by the KU Leuven ethical committee in accordance with the European Communities Council Directive of 22 September 2010 (2010/63/EU) and the Belgian legislation (KB of 29 May 2013).

## Author contributions

VM: Conceptualization, Data curation, Formal analysis, Methodology, Visualization, Writing – original draft, Writing – review & editing. IP: Data curation, Writing – review & editing, Formal analysis. CZ: Writing – review & editing. JVH: Writing – review & editing. LA: Conceptualization, Funding acquisition, Supervision, Writing – review & editing.
