# Supplementary material for: Age-related alterations in the behavioral response to a novel environment in the African turquoise killifish (Nothobranchius furzeri)
Source: Front Behav Neurosci. 2024 Jan 8;17:1326674. doi: 10.3389/fnbeh.2023.1326674 (PMC10800983; doi:10.3389/fnbeh.2023.1326674)
Supplement: Supplementary file 1 [file Data_Sheet_1.docx]

Supplementary Material

Age-related alterations in the behavioral response to a novel environment in the African turquoise killifish (*Nothobranchius furzeri*)

Valerie Mariën^1^, Ilayda Piskin^1^, Caroline Zandecki^1,2^, Jolien Van houcke^1^, and Lutgarde Arckens^1,3^*

^1^Laboratory of Neuroplasticity and Neuroproteomics, Department of Biology, KU Leuven, 3000 Leuven, Belgium

^2^Laboratory of Developmental Neurobiology, Department of Biology, KU Leuven, 3000 Leuven, Belgium

^3^Leuven Brain Institute, KU Leuven, 3000 Leuven, Belgium

*** Correspondence:** Lutgarde Arckens, Ph.D., Laboratory of Neuroplasticity and Neuroproteomics, Katholieke Universiteit Leuven, Naamsestraat 59, box 2467 B-3000 Leuven, Belgium, E-mail: [lut.arckens@kuleuven.be](mailto:lut.arckens@kuleuven.be)

**Supplementary Table 1: Mean parameters of behavior analysis over different ages in the open field test.** The standard error of the mean is depicted between brackets. Center avoidance is calculated as the total distance moved in the peripheral zone divided by the total distance moved in the center and periphery, multiplied by 100. At age 9 weeks, two fish were excluded since the recording was too short.

|  | 6w | 9w | 12w | 15w | 18w | 21w | 24w |
| --- | --- | --- | --- | --- | --- | --- | --- |
| Total distance moved (cm) | 3350 (292.6) | 2529 (316.3) | 3382 (352.1) | 3972 (367.0) | 1917 (320.8) | 2772 (388.9) | 1863 (331.9) |
| Mean velocity (cm/s) | 1.88 (0.16) | 1.41 (0.18) | 1.88 (0.20) | 2.21 (0.20) | 1.07 (0.18) | 1.54 (0.22) | 1.04 (0.18) |
| Cumulative duration center zone (s) | 275.7 (30.9) | 465.8 (71.7) | 307.1 (51.9) | 204.7 (33.8) | 671.1 (77.4) | 471.1 (59.4) | 536.0 (60.4) |
| Latency to first visit center (s) | 22.5 (13.1) | 37.8 (20.3) | 18.6 (7.6) | 29.3 (17.0) | 17.9 (5.7) | 24.8 (14.2) | 13.7 (3.8) |
| Center avoidance (%) | 90.4 (1.65) | 81.6 (3.41) | 86.8 (2.78) | 90.8 (1.67) | 66.9 (3.89) | 77.8 (3.3) | 74.6 (3.4) |
| Zone transitions (#) | 78.5 (9.9) | 63 (9.5) | 66.1 (7.6) | 62.1 (8.4) | 75.9 (10.5) | 73.3 (8.9) | 51.4 (6.8) |
| Immobile duration (s) | 958.2 (62.8) | 1146 (71.1) | 948.2 (66) | 837.5 (63.8) | 1360 (65) | 1227 (64.4) | 1411 (62.7) |
| Number of fish (#) | 16 | 14 | 16 | 14 | 14 | 14 | 14 |

Supplementary Table 2: Mean parameters of behavior analysis over different ages in the novel tank diving test. Standard error of the mean is depicted between brackets. Immobility is measured as a velocity <0.1 cm/s for at least 1 sec.

|  | 6w | 9w | 12w | 15w | 18w | 21w | 24w |
| --- | --- | --- | --- | --- | --- | --- | --- |
| Total distance moved (cm) | 3704 (349.8) | 2069 (248.3) | 2257 (296.8) | 2522 (350.8) | 2184 (199.5) | 3093 (352.2) | 3051 (529.0) |
| Mean velocity (cm/s) | 2.08 (0.19) | 1.16 (0.14) | 1.27 (0.17) | 1.43 (0.20) | 1.23 (0.11) | 1.74 (0.19) | 1.71 (0.29) |
| Latency to top (s) | 173.2 (79.9) | 118 (96.3) | 178.3 (78.4) | 159.5 (68.5) | 226.1 (90) | 157.4 (66.8) | 43.7 (20.9) |
| Time spent in bottom half (%) | 76.32 (5.1) | 82.84 (3.5) | 89.48 (3.2) | 74.7 (7.2) | 80.11 (5.3) | 72.80 (5.8) | 81.97 (4.8) |
| Immobility bouts (#) | 218.6 (16) | 294.3 (21.5) | 266.2 (18.3) | 285 (23.6) | 303.2 (10.8) | 264.3 (18.8) | 254.5 (20) |
| Mean mobility (%) | 8.841 (0.89) | 5.308 (0.72) | 5.179 (0.71) | 5.812 (0.92) | 4.837 (0.52) | 6.69 (0.86) | 3.427 (0.66) |
| Immobility duration (s) | 493.8 (69.5) | 725.9 (68.1) | 815.9 (82.5) | 717.7 (73.3) | 767.4 (58.7) | 562.3 (76) | 670.1 (112) |
| Number of fish (#) | 14 | 14 | 14 | 14 | 14 | 12 | 10 |


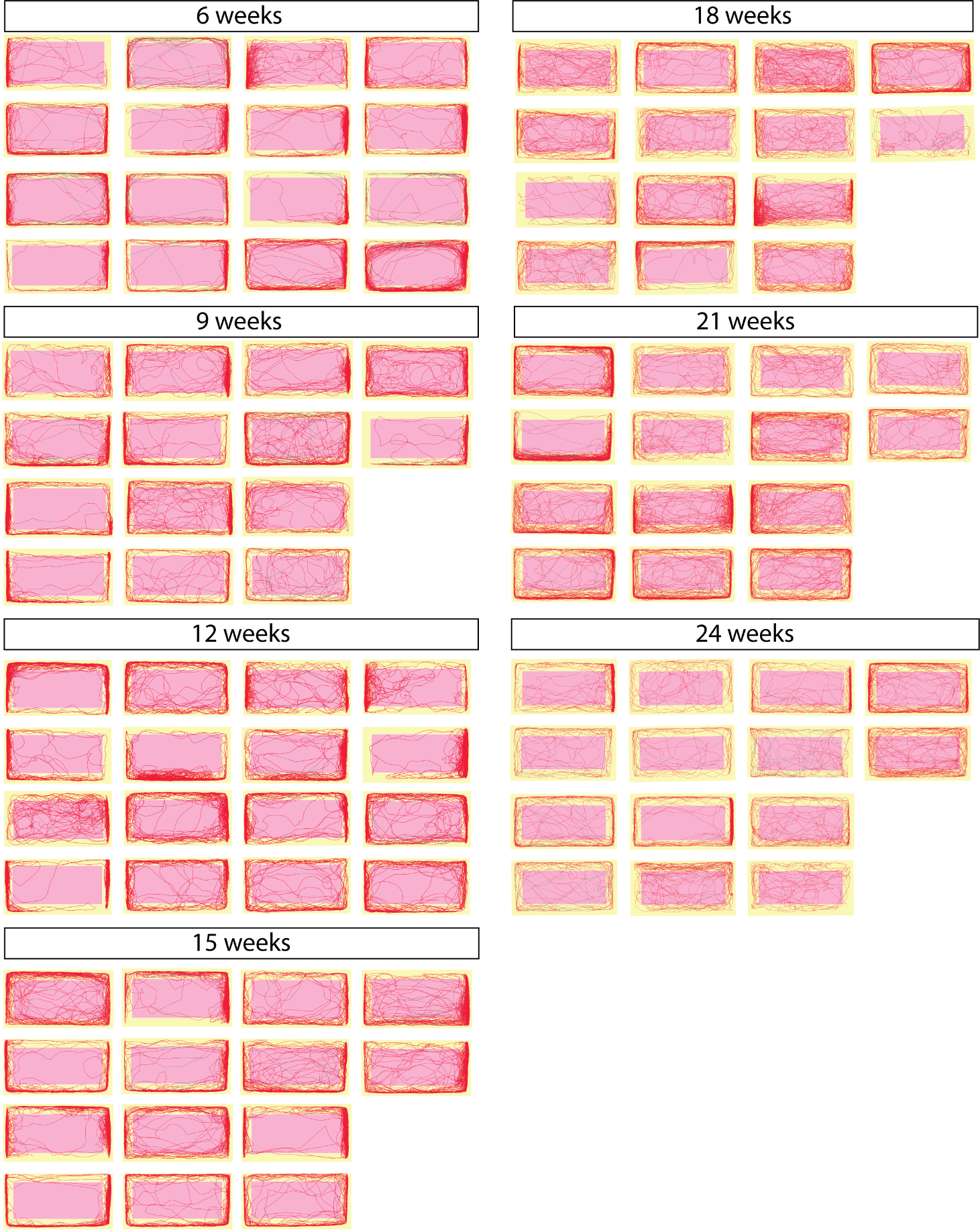


Supplementary Figure 1: All tracks of the different fish in a 30-min. open field test recording. The pink area indicates the center zone, and the yellow area indicates the peripheral zone. Note that the center zone is smaller as the fish are older.


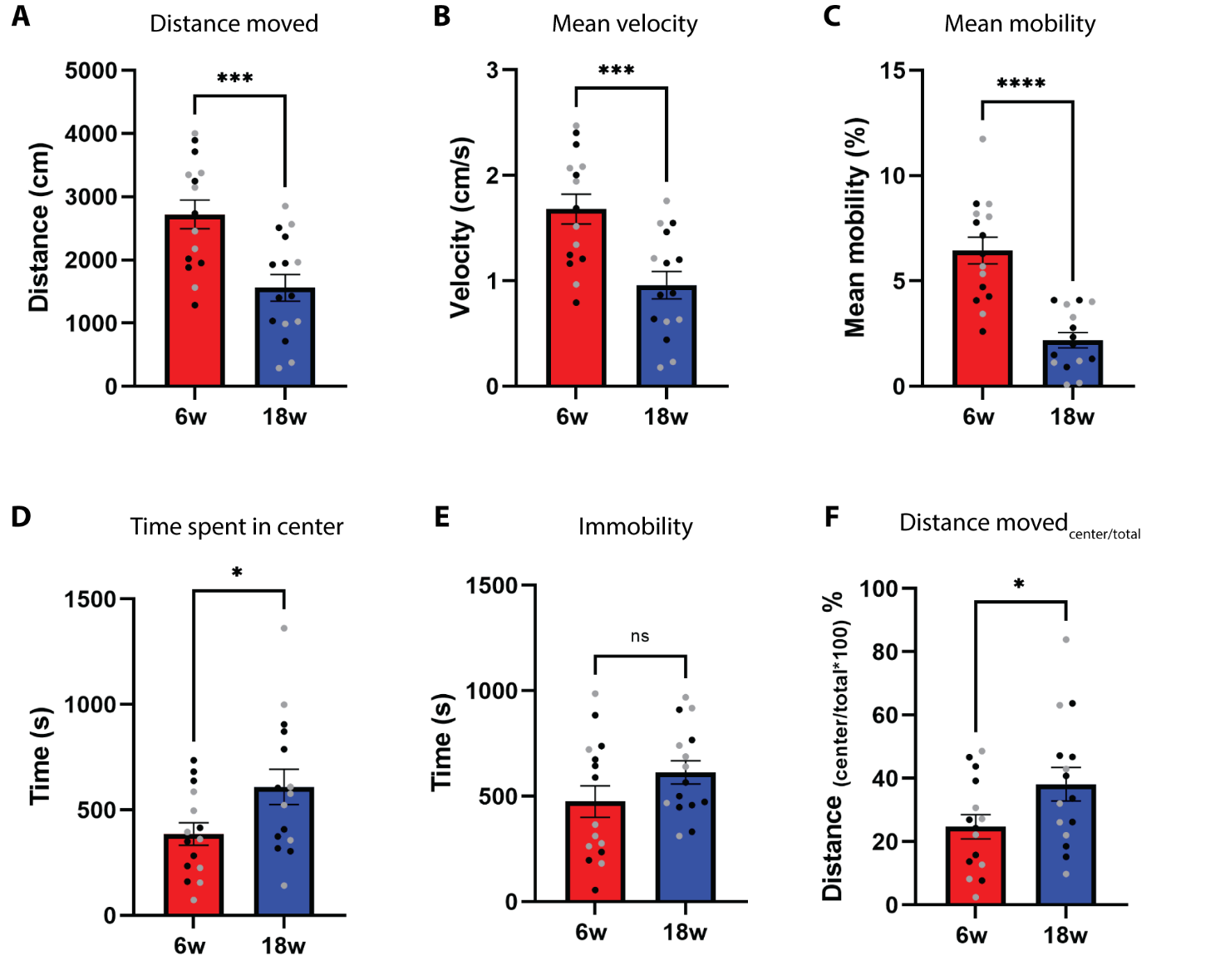


Supplementary Figure 2: Independent open field experiment showing reproducibility of the data. In this experiment, only 6w and 18w old fish were tested in the open field test. Both male (black data points) and female (grey data points) fish were used. After checking for normal distribution, an unpaired t-test was performed. N=15 fish per age group. (A) Total distance moved, (B) Mean velocity, (C) Mean mobility, (D) Time spent in the center zone, (E) Immobility, (F) Distance moved in the center divided by the total distance moved. Immobility is defined as a velocity lower than 0.1 cm/s for a duration of at least 1s. Significant differences are indicated with an asterisk. * p<0.05, *** p<0.001, *** p<0.0001.


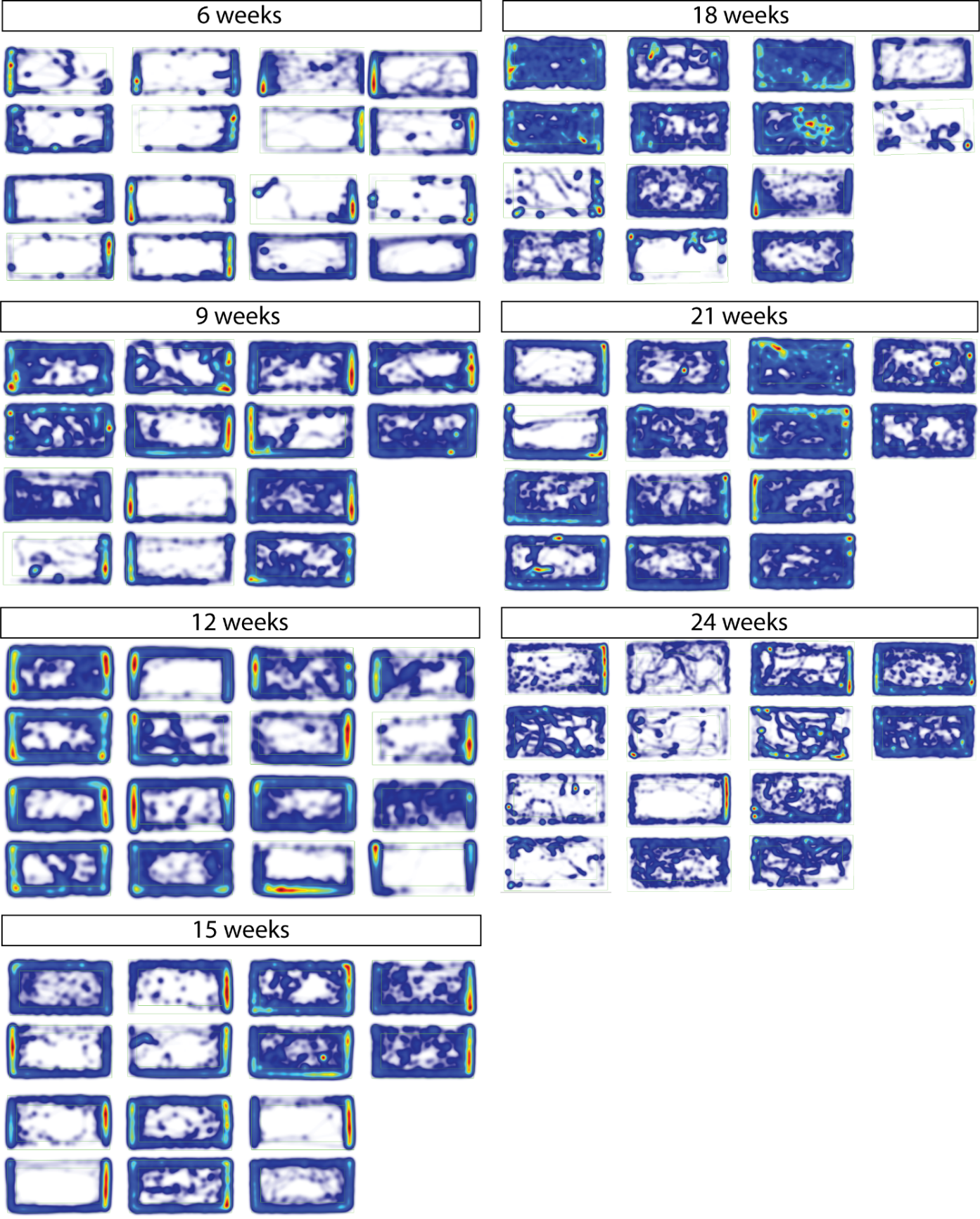


Supplementary Figure 3: All heatmaps of the different 30-min. recordings in the open field test. Red areas indicate the places where fish spent the most of their time.

**
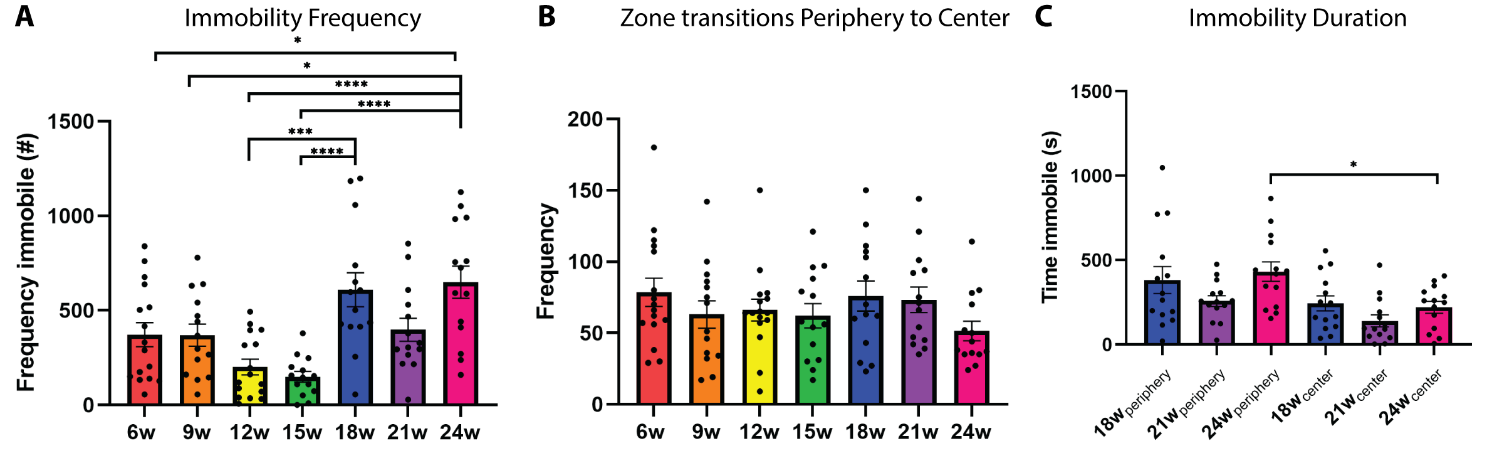
**

Supplementary Figure 4: Immobility frequency (A), zone transitions from the periphery to the center (B) and immobility duration (C) in the open field test for the different fish ages. 30-min. recordings were carried out and fish were observed in an open field test every three weeks from age 6 weeks until age 24 weeks. (A) The immobility frequency increased in the older fish ages (18w, 21w, 24w). (B) Zone transitions from the periphery to the center zone in a 30-min. recording shown for the different ages. No significant differences were observed. (C) The time immobile in the older fish was higher in the peripheral zone than in the center zone (24wperiphery vs. 24wcenter zone, p=0.0118). Points represent individual fish. n=14-16 per age group. w= weeks. Significant differences are indicated with an asterisk. * p<0.05, *** p<0.001, *** p<0.0001.


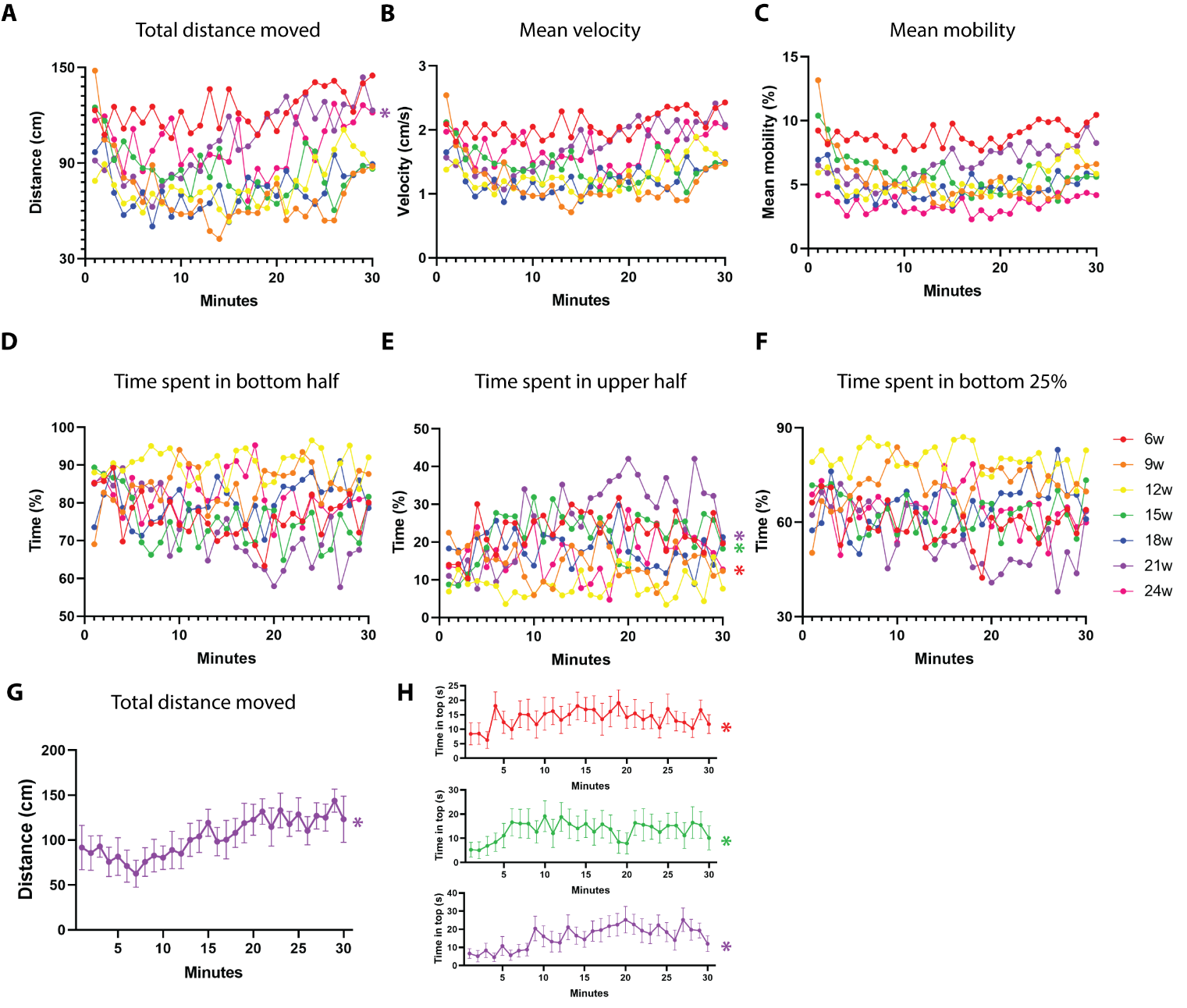


Supplementary Figure 5: Temporal analysis of behavior in the novel tank test did not reveal strong habituation of killifish to the novel tank. (A) Total distance moved, (B) Mean velocity, (C) Mean mobility, (D) Time spent in the bottom half, (E) Time spent in the upper half, (F) Time spent in the bottom 25%, and (G) total distance moved for 21w old fish represented as one minute time bins (H) Habituation was observed for time spent in the upper half for ages 6w, 15w, and 21w. The fish increased the time in the upper half over the 30 min. recording. n=10-14 per age group. w= weeks. Significant differences are indicated with an asterisk. * p<0.05. Points represent the means of the different fish per age group. w=weeks.


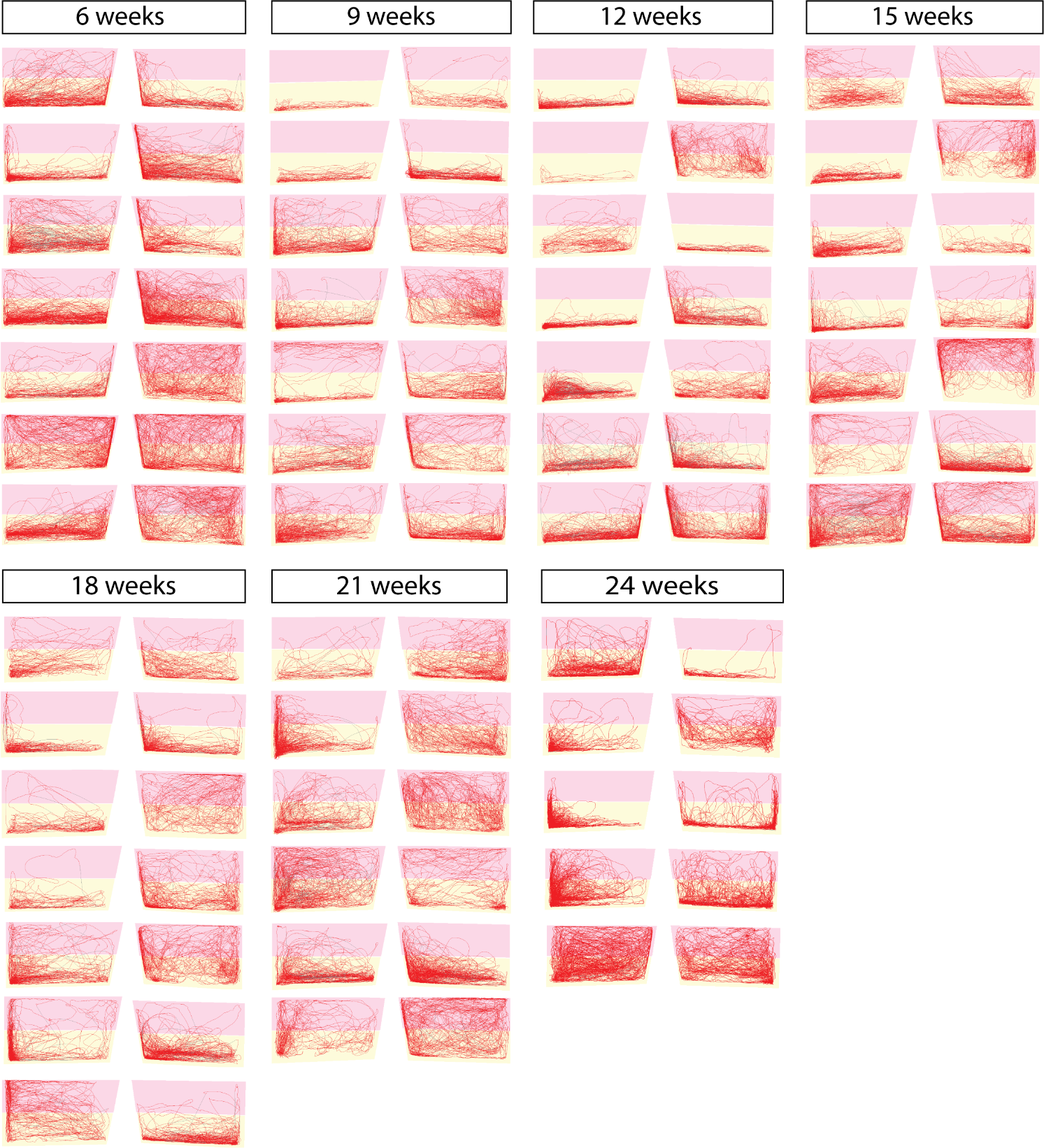


Supplementary Figure 6: Tracks per fish in a 30-min. recording in the novel tank diving test. The pink color indicates the upper half, the yellow color indicates the bottom half.


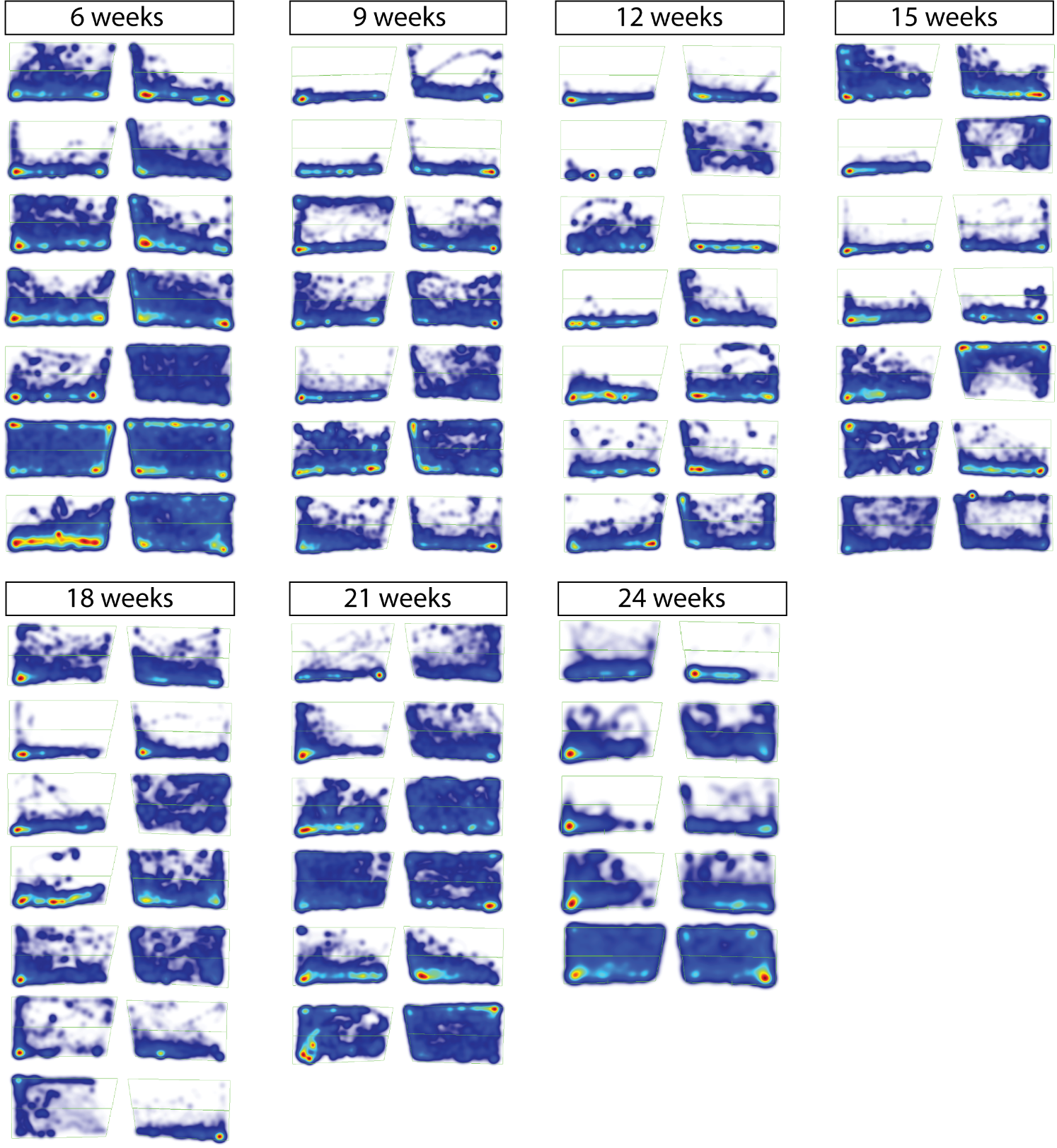


Supplementary Figure 7: Novel tank diving test heatmaps per fish in a 30-min. recording in the novel tank diving test. The red color indicates areas where fish spent the most time.
